# The Influence of Available Cu and Au Nanoparticles (NPs) on the Survival of Water Fleas (*Daphnia pulex*)

**DOI:** 10.3390/ijerph16193617

**Published:** 2019-09-26

**Authors:** Małgorzata Garncarek, Monika Kowalska-Góralska, Magdalena Senze, Katarzyna Czyż

**Affiliations:** 1 Department of Hydrobiology and Aquaculture, Wrocław University of Environmental and Life Sciences, 50-375 Wrocław, Poland; garncarek.gosia@gmail.com (M.G.); magdalena.senze@upwr.edu.pl (M.S.); 2 Institute of Animal Breeding, Wroclaw University of Environmental and Life Sciences, 50-375 Wroclaw, Poland; katarzyna.czyz@upwr.edu.pl

**Keywords:** nano-copper, nano-gold, ecotoxicology, zooplankton

## Abstract

Applications of nanotechnologies in different sectors and everyday items are very promising and their popularity continues to grow. The number of products containing nanoparticles makes environmental exposure to nanoparticles inevitable. The current understanding of the relationships between nanoparticles and the environment is inadequate despite the fast growth of nanotechnologies. The aim of the study was to investigate the influence of copper and gold nanoparticles on *Daphnia pulex* survival. Our study included 48-h acute toxicity tests and determination of median lethal concentration values (LC_50_s) for Cu-NPs and Au-NPs. For nano-copper, 24 h LC_50_ was assumed > 1 mg/L, and 48 h LC_50_ = 0.5117 mg/L. For nano-gold the LC_50_ value after 24 h was 0.4027 mg/L, and after 48 h 0.1007 mg/L. The toxicity of nano -gold solutions was thus found to be higher than that of nano-copper. The addition of Cu-NPs at 0.0625 mg/L and 0.125 mg/L caused an increased multiplication of daphnia, while Au-NPs at 1 mg/L was an inhibitor of reproduction.

## 1. Introduction

A nanoparticle is defined as a small particle of matter with dimensions from one to one hundred nanometers. The presence of nanoparticles (NPs) in nature has been observed for a long time, because they are formed during combustion processes, such as fires or volcanic eruptions [1]. They are also found in fogs and fumes, and are also formed during welding. Recently, humans have deliberately generated NPs. Due to the distinctive optical, magnetic and catalytic characteristics of nanomaterials compared to natural substrates, nanoparticles are being produced on a huge scale [2,3]. The difference in properties is due to the possibility to synthesize nanoparticles with precise control over the size, shape and purity of NPs [4,5,6,7]. Today, nanotechnology is a comparatively young field with its roots dating back to the 1950s. There has been a steady increase in the amount of nanotechnology-based products available on the market, so environmental exposure to NPs is inevitable [8]. Solutions that allow nanoparticles to be used in various sectors are comparatively recent, so there are no extensive, long-term studies to demonstrate what effect they can have on organisms’, including humans’, health and life.

Nanocolloids are growing in popularity on the market. They are widely available and can be purchased without leaving home, e.g. via the Internet. There is no need of prescription to purchase them, and before applying, there are no recommendations for consulting a doctor or other expert. Nanocolloid manufacturers claim many positive benefits for people, while they do not report any contraindications to their use or environmental hazards. 

According to a manufacturer of colloidal solutions of nano-copper and nano-gold, the flat structure of colloid molecules makes their adhesion surface many fold larger than that of similar preparations with particles of spherical structure. In addition, the manufacturer provides the size of gold particles in solution, the diameter of which does not exceed 3 nm. Commercially available solutions are highly concentrated: nano-platinum is available at a concentration of 10 mg/L, nano-copper and nano-gold at a concentration of 25 mg/L and nano-silver at a concentration of 50 mg/L (https://www.vitacolloids.pl/).

The annual worldwide production of Cu-NPs and CuO-NPs is estimated at 200 tons [9] of the products in different concentrations. Among the nanocolloids available on the market, copper has the strongest fungicidal [10,11] and antibacterial [12,13,14,15] effects. Non-ionic colloids of nano-copper can be used to improve the appearance of skin and hair, and against fungal infections in foot care. Additionally, they supports the fight against free radicals by slowing down the skin ageing process [16]. Nano-copper is also used in deodorants as it reduces excessive sweating and eliminates unpleasant sweat smell. Cu-NPs are used in everyday products such as cosmetics (hair conditioners, body lotions, shower gels and facial mists), computer equipment and dietary supplements (http://nanodb.dk/en/).

The conducted research confirms that the addition of nano-copper to water at a concentration of 10 mg/L inhibits the development of *Candida albicans* fungi after 15 minutes [14]. This suggests that such solutions can be added to swimming pool water. However, research conducted by Llorens et al. in 2012 [17] proved that NP solutions have harmful effects on mammalian cells, causing instability of chromosomes and the induction of apoptosis. According to the literature data, the lowest oral toxic dose to humans is 0.01 mg/kg body weight [18,19], while the lowest oral toxic dose given subcutaneously to rabbits is 375 mg/kg body weight and 3 g/kg when administered orally over 60 days [20].

Copper belongs to the microelements needed by the human body. It activates an enzyme, ceruloplasmin, which is essential for the formation of erythrocytes, thus influencing the proper functioning of the circulatory system [21]. In addition, it oxidizes vitamin C and stimulates the synthesis of collagen and elastin, affecting the regeneration of connective tissue. The copper found in ceruloplasmin is one of the most mobile forms of this element in organisms and in this form it regulates iron metabolism and transport, therefore, its absence contributes to anemia [13,21]. According to the World Health Organization, the recommended dose of copper is 2 mg/day for adults [22]. The richest sources of copper in the human diet are cocoa (3.76 mg Cu per 100 g product), sunflower seeds (1.87 mg), pumpkin seeds (1.57 mg) and hazelnuts (1.29 mg). The richest source of copper among animal products is pork liver—100 g of liver contains 0.63 mg of Cu [23].

Like colloidal copper nanoparticles, nano-gold exhibits antibacterial properties [24,25]. However, unlike copper, gold is not an element that occurs naturally in organisms. Thus, there is no reason to assume that there are physiological symptoms of gold deficiency. According to the available list, The Nanodatabase, Au-NPs are used in everyday products such as cosmetics (body lotions, eye moisturizers, serums and anti-wrinkle elixirs), photographic fluids, hair straighteners, earrings, toothbrushes, clothing (trousers, anti-ageing gloves and eye masks and bathing suits), oil stain removers and air conditioners (http://nanodb.dk/en/). The properties of nano-gold allowed for a wide application in the cosmetic industry due to stimulation of collagen synthesis and inhibition of elastin loss, delaying the appearance of wrinkles. Au-NPs also inhibits skin ageing and helps to combat ageing symptoms, such as age spots. Additionally, it is used to cleanse and regenerate the skin by strengthening the damaged cells of this tissue.

Nano-gold molecules are used in medicine and the pharmaceutical industry. Au-NPs has been successfully used in biomedical imaging [26,27,28] and cancer therapies [29,30]. Nano-gold is used in photothermal therapy [31,32,33,34] and photodynamic therapy [35,36]. Nano-gold molecules are used as drug carriers [37,38,39], including anticancer drugs [40,41] and vaccines [42]. The use of nano-gold drugs has a high potential but is a cause of concern for local and systemic toxicity to living organisms [43]. After entering the body, Au-NPs are metabolized in the liver, where they can accumulate or be transferred other organs (e.g. the brain) via blood vessels [44]. During photodynamic therapy, nano-gold particles cause oxidative stress and contribute to reactive oxygen species (ROS) production [45,46]. It was also observed that the toxicity of nanoparticles of in vitro models differs significantly from that of in vivo models. Au-NPs are considered non-toxic in vitro [47], but the injection of nano-gold particles into mice caused tissue damage and apoptosis in the liver [48] and the injection of nano-gold into rabbits induced apoptosis of cartilage tissue cells [49].

The growing interest in nanotechnology applications in various fields of industry, cosmetics, medicine and everyday life is a reason for a steady increase in their production volume, and consequently also in their emissions to the environment, including the aquatic environment [50]. In addition, the aquatic environment is the ultimate collector of pollutants, including NPs, whether they have been released directly into the water or indirectly, for example through wastewater treatment plants or landfills [43]. Water purity is particularly important for the quantity and richness of water ecosystems. Nanoparticles can interact with other pollutants entering the water, creating compounds that are even more toxic to organisms [51]. Planktonic organisms are the most vulnerable to water pollution and are a key component of aquatic ecosystems, forming the basis of most food networks. The accumulation of xenobiotics (NPs) in water fleas leads to further, higher accumulation in fry (small fish up to the age of one eating zooplankton). Fish, as another element of the food chain, accumulate even more harmful substances. The last organism of each food chain absorbs the largest amount of xenobiotics. In the case of water nets, the last organism may be a human eating fish with accumulated nanoparticles. NPs accumulate in different organs, causing tissue damage [52]. In addition, the toxicity of nanoparticles to zooplankton can reduce its survival and cause the loss of many trophic networks in the aquatic ecosystem [53].

A different dose of NPs may be toxic for different organisms, therefore there is a need to conduct the studies aimed to determine the median lethal concentration LC_50_ for a variety of species. Such studies will make it possible to establish threshold values for a given xenobiotic that cannot be exceeded. This will help to establish standards for the concentrations of nanoparticles used, thereby reducing and slowing down the rate of NPs accumulation in living organisms.

Current knowledge about copper and gold nanoparticles is limited and does not show the full toxic effects on organisms living in aquatic ecosystems. The effect of nano-copper and nano-gold (in the colloidal form available on the market) on zooplankton has not yet been well recognized. Therefore, the influence of aqueous solutions with increasing concentrations of Cu NPs and Au NPs on the model organism—*Daphnia pulex*—was examined in the study presented. 

## 2. Materials and Methods 

The study was carried out on *Daphnia pulex* (water flea) organisms, a species belonging to the genus *Daphnia* of the family *Daphniidae*. Water fleas were placed in the aquarium and cultured before the experiment. Solutions for the study were prepared from stock solutions of non-ionic colloidal nano-copper at 25 mg/L and non-ionic colloidal nano-gold at 25 mg/L purchased from the Vitacolloids company (vitacolloids.pl), manufactured using physical methods.

A dilution series of Cu-NPs and Au-NPs solutions from 0 to 1 mg/L were made, the control solution was tap water. Modified 48-h acute toxicity tests were performed on 6-well plates. 10 ml of the appropriate solution was poured into each well. Well number 1 was the control, well number 2 was the 0.0625 mg/L solution, well number 3 was the 0.125 mg/L solution, well number 4 was the 0.25 mg/L solution, well number 5 was the 0.5 mg/L solution and well number 6 was the 1 mg/L solution. The procedure was the same for plates with nano-copper and nano-gold solutions. 

The modification of the toxicity test included testing of mature *Daphnia pulex*, which better reflects environmental conditions than testing substance effects on juveniles. Five daphnias were transferred to each well using a sterile Pasteur pipette. The plates were stored in a shaded place and opened only when counting daphnia after 24 and 48 h of incubation in solutions. The culture medium was aquarium water the culture was conducted in, and the light dark cycle was 16 h/8 h. The experiment was performed five times, with three repetitions each time, giving a total of 15 repetitions for each concentration of the solution of nano-copper and nano-gold.

The results were compiled in MS Excel spreadsheet. The mortality percentage was calculated using the equation:

(1) $$Mortality\;rate=\frac{deaths\;occurring\;within\;a\;given\;time\;period}{size\;of\;the\;population\;in\;which\;the\;deaths\;occur}\cdot 100\%$$

The mean and standard deviation of the tests was calculated using the R software (ver. 3.4.4) (https://www.r-project.org/). The K deviation rate has been calculated from:

(2) $$K=\frac{50-P1}{P2-P1}$$

where P1 is a mortality rate of less than 50% (closest 50%) and P2 is a mortality rate of more than 50% (closest 50%). The LC_50_ (lethal concentration) value, i.e., the concentration value at which 50% of the examined population dies, was calculated from the formula:

(3) $$\log LC_{50}=\log x+K\cdot \log i$$

where *x* represents the lower and closest concentration to 50% mortality and *i* is the ratio of the geometric rate (*i*=2). Statistical differences between % mortality rate measured at 24 or 48 h, respectively, were evaluated using Kruskall-Wallis and post-hoc tests at a significance level of *p* < 0.05 (R software ver. 3.4.4).

## 3. Results

The observed change of daphnia number as a result of 24- and 48-h incubation in solutions with different concentrations of copper and gold nanoparticles is presented in Table 1. 

**Figure 1:**
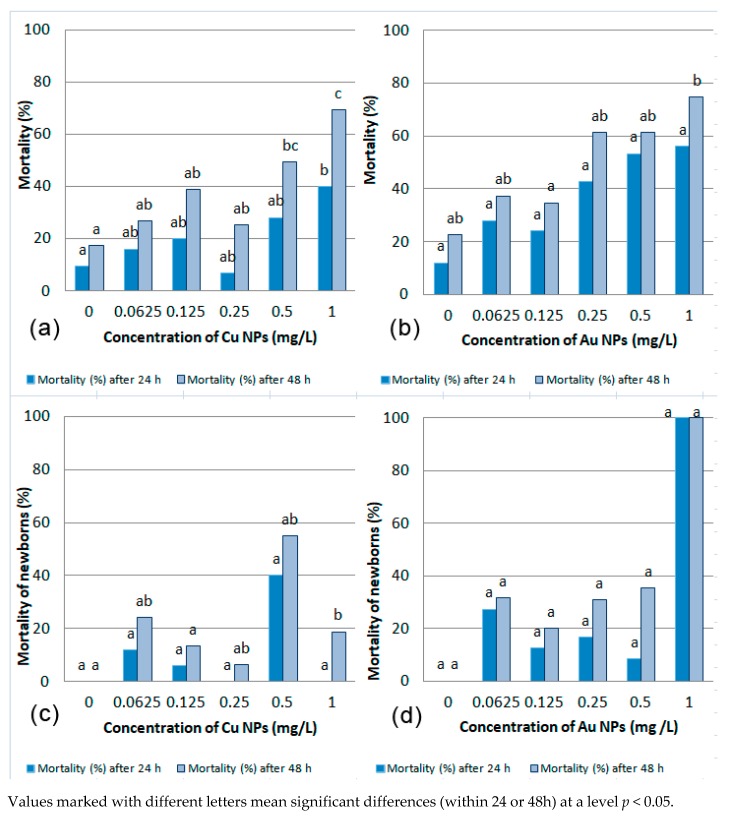
*Daphnia pulex* mortality (%) of depending on the concentration of Cu NPs (**a**); Au NPs (**b**); newborns *Daphnia pulex* depending on the concentration of Cu NPs (**c**) and Au NPs (**d**).

The most toxic among nano-copper solutions was the dose of 1 mg/L. The dose of 0.25 mg/L caused the lowest average mortality of *Daphnia pulex* after both 24 h and 48 h. In this study group there were fewer dead daphnia after 24 h than in the control sample without the addition of nano-copper. For 48-h incubation of *Daphnia pulex* the LC_50_ value was calculated as 0.5117 mg/L. The percentage mortality of *Daphnia pulex* depending on nano-copper concentration and incubation time is shown in Figure 1a.

Mature daphnias were multiplying during incubation in Cu-NPs solutions. The number of newborn daphnias after 24 and 48 h of incubation depending on the concentration of copper nanoparticles and the effect of nano-copper on newborn daphnias is presented in Table 2. 

The highest number of newborn daphnias was observed in solutions with Cu-NP concentrations at the level of 0.0625 mg/L and 0.125 mg/L. More newborn daphnias were noted in these concentrations of solutions than in the control, which indicates that small doses of nano-copper cause increased reproduction of daphnias. The most toxic concentration of Cu NPs was 0.5 mg/L. Figure 1c shows the relationship of mortality rates of newborn *Daphnia pulex* and the concentration of nano-copper in solutions. After 24-h incubation of newborn daphnias, the *LC*_50_ value could not be calculated, as there was no mortality higher than 50% at any concentration. After 48-h incubation, the *LC*_50_ value for newborns is 0.1117 mg/L (Table 3). However, this value is not an accurate *LC*_50_, as not all individuals have been exposed to nano-copper for 48 h. Some of them multiplied during the first day of the study, and some only between 24 and 48 h of observation, so it is impossible to determine at what point in time each newborn daphnia appeared.

The least toxic dose of nano-gold is 0.125 mg/L, both after 24 h and 48 h incubation. Nano-gold is most toxic to daphnia at a concentration of 1 mg/L, regardless of incubation time. After 24-h incubation in solutions with different concentrations of nano-gold, it was possible to calculate the LC_50_, which was 0.4027 mg/L. For 48-h incubation of daphnia in solutions with Au-NPs LC_50_ = 0.1007 mg/L (Table 3). Figure 1b shows the changes in the number of daphnia (in percentage of mortality) caused by the addition of different concentrations of colloidal nano-gold solution to water after 24 and 48 h of incubation.

During incubation in the Au-NPS solutions mature daphnias were multiplying. The number of newborn daphnias after 24 and 48 h of incubation depending on the concentration of gold nanoparticles and the effect of nano-gold on newborn daphnia is presented in Table 2. After 24-h incubation in a nano-gold solution the least toxic dose was 0.5 mg/L, and after 48 h it was 0.125 mg/L. Only one daphnia was observed at Au-NPs 1 mg/L sample and it did not survive at this concentration. This also shows that the addition of nano-gold at a concentration of 1 mg/dm^−3^ is an inhibitor of daphnia reproduction. The percentage of mortality at different Au NPs concentrations is shown in Figure 1d. The mortality rate for 1 mg/L concerns only one individual whose death may be related both to the toxicity of that concentration and to the production of the individual particularly sensitive to nano-gold. For newborn daphnia during the first day of incubation in nano-gold solutions, the LC_50_ value is 0.0776 mg/L. For newborn occurred during 48 h of study, the LC_50_ value is 0.5853 mg/L (Table 3). 

## 4. Discussion

Due to increasingly common release of nanoparticles into the environment, studies on their toxicity are necessary. Research shows that nanoparticles can be environmentally risky due to their small size, large active surface area and ability to produce reactive oxygen species [54].

The mortality rate was below 50% after a 24-h incubation with nano-copper solutions in each of the tested samples, so the LC_50_ is certainly higher than 1 mg/L. Our results are consistent with previously published studies on the influence of CuO NPs on *Daphnia magna*, which indicate the value of LC_50_ = 2.5578 mg/L [55] or even LC_50_ = 7.85 mg/L [56]. After a 24-h incubation of the *Daphnia pulex* in nano-copper solutions it can be assumed that water flea has a defense system that allows it to survive at a concentration of 0.25 mg/L better than in the control sample. After 48-h incubation of the water flea in solutions with different concentrations of nano-copper, LC_50_ was 0.5117 mg/L. It proves that the toxicity of nanoparticles increases with increasing exposure time and that the toxicity of Cu-NPs is correlated with incubation time. It is confirmed in the study by Khoshnood et al. [56] in which LC_50_ for *D. magna* after 24 h of CuO NPs incubation was 7.85 mg/L, and after 48 h of incubation it was LC_50_ = 6.62 mg/L.

The first toxicity study of CuO nanoparticles for *Daphnia magna* was conducted in 2008, and the LC_50_ after 48 h of incubation was 3.2 mg/L, while for copper ions from CuSO_4_ LC_50_ solution it was only 0.07 mg/L [57]. It proves that nano-copper solutions are less harmful to the aquatic environment than copper ions. Further studies with the use of CuO NPs solutions for *D. magna* indicate the LC_50_ value at the level of 3.3 mg/L [58], 0.99 mg/L [59], 5.66 mg/L [60] and 5.9 mg/L [61]. Research has shown that nano-copper solutions can be harmful to the aquatic environment due to more ecotoxic copper ions [57,62,63,64,65,66] released from the solution. Cu NPs does not dissolve completely [67], however, it is still a source of copper ions. Greater harmfulness of copper ions from CuSO4 solutions was confirmed in the studies in which LC_50_ was 0.019 mg/L [68], 0.00653 mg/L [69], 0.045 mg/L [11] and 0.07 mg/L [58]. LC_50_ values for *Daphnia pulex* are much lower than LC_50_ values for *Daphnia magna* and this is most likely due to the difference in size between the two species (*D. pulex* is almost twice smaller), so that *D. magna* can take a higher dose of nano-copper.

The conducted studies suggest that low concentrations (0.0625 mg/L and 0.125 mg/L) of nano-copper may enhance daphnia reproduction. Higher number of multiplied daphnia can be observed at low concentrations than in the control for both 24-h and 48-h incubation. This may be related to the fungicidal and antibacterial properties of the nanoparticles [10,12], which provide better conditions for mature daphnia to reproduce. Also under the influence of a stress factor such as the addition of a small amount of Cu NPs, survival is more important for daphnia than keeping the offspring alive, which may explain why there are more newborn daphnias in these concentrations compared to the control. The inhibition of daphnia reproduction by copper has been demonstrated in a study by Taylor et al. [70], where the addition of copper ions to water at a concentration of 1 µg/L inhibited reproduction by 9% and the addition of copper ions at a concentration of 1.8 µg/L reduced reproduction by 30% compared to the control sample. This may be an explanation for the lack of renewal of daphnia population in lakes heavily contaminated with copper ions.

After 24-h exposure of mature *Daphnia pulex* to solutions with addition of nano-gold the LC_50_ amounted to 0.4027 mg/L, and after 48-h exposure the LC_50_ amounted to 0.1007 mg/L. The most toxic concentration causing death of 56% of individuals after the first day of incubation and about 75% after the second day of incubation was 1 mg/L. The literature data show LC_50_ values for *Daphnia magna* incubated with nano-gold solution for 48 h as 0.32 mg/L [60] and 0.5 mg/L [71]. A study by Li et al. [72] suggested a much higher value of LC_50_ than in other studies for *Daphnia magna*, and its value was estimated at about 70 mg/L. LC_50_ values for *Daphnia pulex* are much lower than LC_50_ values for *Daphnia magna*, most likely due to the difference in size between the two species (*D. pulex* is almost twice smaller), so that *D. magna* can uptake a higher dose of nano-gold. This shows that if there are differences between these closely related species, the LC_50_ should be marked not only on model organisms, but on all those which are exposed to a potential risk of contamination.

The highest number of newborn daphnias after 24-h incubation in Au-NPs solutions was noted at concentrations of 0.25 mg/L and 0.5 mg/L. 24 h LC_50_ for young daphnias was 0.0776 mg/L. After 48-h incubation, the LC_50_ was 0.5853 mg/L. Incubation of water flea with the addition of nano-gold at a concentration of 1 mg/L effectively inhibits the reproduction of daphnia. Studies done on another organism living in water—*Danio rerio—*indicated the harmful effect of nano-gold on hatching and growth of juveniles. Au-NPs at a concentration of 2.5 mg/L reduces the hatching of young *D. rerio* to only 20%, moreover, the young incubated in a solution of nano-gold are apparently smaller than the control group [60]. Toxicity of nano gold is confirmed in the study by Kim et al. [73] in which the LC_50_ after five days of *Danio rerio* incubation was 30 mg/L. This concentration causes abnormal pigmentation and anomalies in eye development during embryonic development of *D. rerio*—a defect that occurred in 80% of young individuals. However, there are studies contrary to those mentioned above. These studies do not indicate any toxicity of Au-NPs to *Danio rerio* [74], which may be related to the fact that only a very low concentration range (0–100 µg·dm^−3^) of Au-NPs was tested.

Literature research proves that gold ions are more toxic compared to nano-gold [44,75]. This was confirmed in the studies in which LC_50_ value after 48-h incubation of *Daphnia magna* in solutions with addition of gold ions (derived from HAuCl_4_) was 2 mg/L, while LC_50_ value for nano-gold was about 70 mg/L [72]. In case of *Daphnia pulex,* 48 h LC_50_ for nano-gold was 7.31 mg/L and for gold ions it was LC_50_ = 0.01 mg/L [76]. Due to the toxicity revealed by nano-gold particles, there are studies using Au-NPs coating substances, which reduce their toxicity [73,77,78,79]. The size of nano-gold molecules in solutions is another factor affecting its ecotoxicity, but compared to coatings it is a secondary issue [80]. Studies have proven higher toxicity for smaller (10–12 nm diameter) than larger (50 nm diameter) nano-gold particles [46,81,82].

To sum up, it was found on the basis of the conducted study that nano-copper and nano-gold particles are toxic to *Daphnia pulex*. Compared to the literature data, the study results show that the toxicity of nano-copper and nano-gold is different depending on the species of organism. The variability in the range of LC_50_ values both for nano-copper and nano-gold reported in the literature may be due to differences in the experimental designs or features like particle size and stabilizing agent used. However, it can be concluded that in order to reduce the ecotoxicity of Cu NPs and Au NPs, it is recommended to use very low concentrations (not exceeding the LC_50_) of solutions or, according to literature data, to conduct studies to coat nanoparticles with other chemical substances, thanks to which the solutions of nanoparticles will become safer for the environment.

## 5. Conclusions

The values of lethal concentrations after the influence of nanoparticles on *Daphnia pulex* were: for 24 h LC_50_ > 1 mg/L, for 48 h LC_50_ = 0.5117 mg/L. The toxicity of nano-copper increases with increasing exposure time.

Low concentrations (0.0625 mg/L and 0.125 mg/L) of nanoparticles cause increased reproduction of water flea.

The received LC_50_ values for *Daphnia pulex* incubated in nano-gold solutions were: 24 h LC_50_ = 0.4027 mg/L, 48 h LC_50_ = 0.1007 mg/L. The toxicity of nano-gold increases with time of exposure.

The addition of nano-gold at a concentration of 1 mg/L is an inhibitor of *Daphnia pulex* reproduction.

Based on LC_50_ values and observations of the number of multiplied water fleas in the conducted studies, it was proved that nano-gold solutions are more toxic than nano-copper solutions. Further studies on the ecotoxicity of nano-copper and nano-gold should be directed to work on reducing the toxicity of substances by using employing appropriate particle sizes and coating agents.

## Figures and Tables

**Table 1:** Effects of different concentrations of NPs solution on survival of *Daphnia pulex.*

NPs	Concentration (mg/L)	Dead after 24 h		Dead after 48 h	
		Mean ± SD	Range	Mean ± SD	Range
Cu	0	0.4667 ± 0.64	(0–2)	0.8667 ± 0.834	(0–2)
	0.0625	0.8 ± 1.014	(0–3)	1.3333 ± 1.345	(0–4)
	0.125	1 ± 1.363	(0–5)	1.9333 ± 1.387	(0–5)
	0.25	0.3333 ± 0.617	(0–2)	1.2667 ±1.033	(0–4)
	0.5	1.4 ± 1.682	(0–5)	2.4667 ± 1.598	(0–5)
	1	2 ± 2.07	(0–5)	3.467 ± 1.302	(0–5)
Au	0	0.6 ± 0.91	(0–3)	1.133 ± 1.356	(0–5)
	0.0625	1.4 ± 1.639	(0–5)	1.867 ± 1.552	(0–5)
	0.125	1.2 ± 1.521	(0–5)	1.733 ± 1.792	(0–5)
	0.25	2.133 ± 1.885	(0–5)	3.067 ± 1.831	(0–5)
	0.5	2.667 ± 2.289	(0–5)	3.067 ± 2.219	(0–5)
	1	2.8 ± 2.274	(0–5)	3.733 ± 1.792	(0–5)

**Table 2:** Effects of different concentrations of NPs solution on survival of newborn *Daphnia pulex.*

NPs	Concentration (mg/L)	Number of Newborns after 24 h	Dead after 24 h		Number of Newborns after 48 h	Dead after 48 h	
			Mean ± SD	Range		Mean ± SD	Range
Cu	0	9	0 ± 0	(0–0)	17	0 ± 0	(0–0)
	0.0625	17	0.133 ± 0.516	(0–2)	29	0.467 ± 0.915	(0–3)
	0.125	17	0.067 ± 0.258	(0–1)	30	0.267 ± 0.458	(0–1)
	0.25	6	0 ± 0	(0–0)	16	0.067 ± 0.258	(0–1)
	0.5	5	0.133 ± 0.352	(0–1)	20	0.733 ± 0.961	(0–3)
	1	7	0 ± 0	(0–0)	16	0.2 ± 0.414	(0–1)
Au	0	7	0 ± 0	(0–0)	31	0 ± 0	(0–0)
	0.0625	11	0.2 ± 0.561	(0–2)	19	0.4 ± 0.737	(0–2)
	0.125	8	0.067 ± 0.258	(0–1)	30	0.4 ± 0.632	(0–2)
	0.25	12	0.133 ± 0.352	(0–1)	26	0.533 ± 0.915	(0–3)
	0.5	12	0.067 ± 0.258	(0–1)	17	0.4 ± 0.507	(0–1)
	1	1	0.067 ± 0.258	(0–1)	1	0.067 ± 0.258	(0–1)

**Table 3:** LC_50_ values calculated for *Daphnia pulex* after 24 and 48 h incubation.

NPs	Developmental Stage	LC_50_ (mg/L)	
		After 24 h	After 48 h
Cu	mature	-	0.5117
	juvenile	-	0.1117
Au	mature	0.4027	0.1007
	juvenile	0.0776	0.5853
